# Exploring the role of two interacting phosphoinositide 3-kinases of *Haemonchus contortus*

**DOI:** 10.1186/s13071-014-0498-2

**Published:** 2014-11-12

**Authors:** Fa-Cai Li, Robin B Gasser, James B Lok, Pasi K Korhonen, Yi-Fan Wang, Fang-Yuan Yin, Li He, Rui Zhou, Jun-Long Zhao, Min Hu

**Affiliations:** State Key Laboratory of Agricultural Microbiology, Key Laboratory of Development of Veterinary Diagnostic Products, Ministry of Agriculture, College of Veterinary Medicine, Huazhong Agricultural University, 1 Shizishan Street, Wuhan, 430070 Hubei China; Faculty of Veterinary and Agricultural Sciences, The University of Melbourne, Corner of Flemington Road and Park Drive, Parkville, VIC 3010 Australia; Department of Pathobiology, School of Veterinary Medicine, University of Pennsylvania, 3800 Spruce Street, Philadelphia, PA19104 USA

**Keywords:** Parasitic nematode, *Haemonchus contortus*, *age-1*, *aap-1*, Development, Transgenesis

## Abstract

**Background:**

Phosphoinositide 3-kinases (PI3Ks) are relatively conserved and important intracellular lipid kinases involved in signalling and other biological pathways. In the free-living nematode *Caenorhabditis elegans*, the heterodimeric form of PI3K consists of catalytic (AGE-1) and regulatory (AAP-1) subunits. These subunits are key components of the insulin-like signalling pathway and play roles in the regulation of the entry into and exit from dauer. Although, in parasitic nematodes, similar components are proposed to regulate the transition from free-living or arrested stages to parasitic larvae, nothing is known about PI3Ks in relation to the transition of third-stage larvae (L3s) to parasitism in *Haemonchus contortus*.

**Methods:**

An integrated molecular approach was used to investigate *age-1* and *aap-1* of *H. contortus* (*Hc-age-1* and *Hc-aap-1*) in *C. elegans.*

**Results:**

The two genes *Hc-age-1* and *Hc-aap-1* were transcribed in all life stages, with the highest levels in the egg, infective L3 and adult female of *H. contortus*. The expression of these genes was localized to the intestine, contrasting the pattern of their orthologues in *C. elegans* (where they are expressed in both head neurons and the intestine). The yeast two-hybrid analysis demonstrated that the adaptor-binding domain of *Hc-*AGE-1 interacted strongly with the *Hc-*AAP-1; however, this complex did not rescue the function of its orthologue in *age-1*-deficient *C. elegans*.

**Conclusions:**

This is the first time that the PI3K-encoding genes have been characterized from a strongylid parasitic nematode. The findings provide insights into the role of the PI3K heterodimer represented by *Hc-age-1* and *Hc-aap-1* in the developmental biology of *H. contortus*.

**Electronic supplementary material:**

The online version of this article (doi:10.1186/s13071-014-0498-2) contains supplementary material, which is available to authorized users.

## Background

Phosphoinositide 3-kinases (PI3Ks) are relatively conserved and important intracellular lipid kinases that synthesize lipid second messengers by phosphorylating the 3-hydroxyl group of the inositol head-group in the phosphatidylinositol and phosphoinositides [1,2]. This phosphorylation process gives rise to the activation of a series of downstream effector proteins, such as protein kinase, regulators of small GTPases and/or scaffolding proteins [3,4], which play key roles in lipid and cell signalling as well as membrane trafficking. In multicellular organisms, including the *Caenorhabditis elegans* (free-living nematode), *Drosophila melanogaster* (vinegar fly) and *Mus musculus* (mouse), PI3Ks play key roles in the regulation of cellular metabolism and/or growth [5-9]. Additionally, PI3Ks are involved in the regulation of human maladies, such as heart disease and cancer, and are thus considered possible therapeutic targets [10].

Based on sequence homology and lipid substrate preference, PI3Ks are classified into classes I to III [11]. In *C. elegans*, PI3K (class I) is a heterodimer consisting of two subunits encoded by *age-1* (orthologue of the p110 catalytic subunit) and *aap-1* (orthologue of the p50/p55-like regulatory subunit) [12,13]. This AGE-1/AAP-1 heterodimer is a key component of the canonical insulin-PI3K signalling pathway, which plays an important role in dauer formation in *C. elegans* [14]; genetic screens have shown that PI3K is downstream of the insulin-like receptor DAF-2 [15-17] and upstream of PDK-1 [18,19], AKT-1/AKT-2 kinases [20] and the fork-head transcriptional factor DAF-16 [21-23]. In *C. elegans*, loss-of-function mutations in the gene *age-1* lead to dauer constitutive phenotypes [6]. Similarly, *C. elegans* lacking AAP-1 have phenotypes that are similar to *age-1* mutants [13]. Upstream signalling via DAF-2 activates PI3K by phosphorylation, which, in turn, negatively regulates DAF-16, a crucial regulator of dauer formation, with its characteristics of developmental arrest, longevity and stress-resistance [24,25].

Although advances have been made in understanding the mechanisms involved in the entry into and exit from dauer in *C. elegans*, very little is known about such mechanisms in parasitic nematodes. Some workers [26-29] have proposed that infective third-stage larvae (L3s) of parasitic nematodes represent the same or a similar developmental state to dauer in *C. elegans*, with comparable features (including an inability to feed, resistance to stress/environment and extended lifespan). In addition, there are indications that mechanisms controlling the transition of free-living to invasive L3s in parasitic nematodes are similar to those that regulate the recovery from dauer in *C. elegans*. For instance, recently, two orthologues of *Ss-age-1* and *Ss-aap-1* were identified in the parasitic nematode *Strongyloides stercoralis* [30]. The proteins and anatomical expression patterns of AGE-1s from *C. elegans* and *S. stercoralis* were shown to be similar. Additionally, LY294002 (a specific inhibitor of PI3-kinase) was shown to block L3 activation *in vitro*, with a cessation of feeding in treated L3s [30,31]. These findings suggested that insulin-like signalling is conserved and plays a critical role in activating L3s of parasitic nematodes during their invasion of the animal host.

The nuclear genomes and transcriptomes of *Haemonchus contortus* [32,33] provide a foundation to begin to explore mechanisms of key molecules involved in developmental processes. However, a lack of effective *in vitro*-culture and genetic methods for parasitic nematodes has hampered the study of the developmental biology of these nematodes [28,34-36]. In contrast, *C. elegans* provides a powerful surrogate system to shed light on gene function in parasitic nematodes, such as *Ancylostoma caninum* [37,38], *H. contortus* [39,40] and *S. stercoralis* [41,42].

In the present study, we isolated and characterized the *C. elegans age-1* and *aap-1* orthologues of *H. contortus* (*Hc-age-1* and *Hc-aap-1*). Using *in vivo* and *in vitro* techniques, we characterized and compared the transcriptional profiles and expression patterns of *Hc-age-1* and *Hc-aap-1* in different developmental stages of the parasite. To investigate the proposed orthology of *Hc*-AGE-1 and *Ce*-AGE-1, we tested whether the adaptor-binding domains of *Hc*-AGE-1 and *Hc*-AAP-1 interacted with each other as a heterodimer in a yeast two-hybrid assay, and then assessed function using constructs containing the *Ce-age-1* and *Hc-age-1* regulatory and coding regions by attempting heterologous rescue of an *age-1* mutant strain of *C. elegans*.

## Methods

### Ethics statement

All the experimental animals used were treated strictly in accordance with the recommendations in the Guide for the Regulation for the Administration of Affairs Concerning Experimental Animals of P. R. China. The protocol employed was approved by the Animal Ethics Committee of Hubei Province (permit no. SYXK-0029). The care and maintenance of animals were in accordance with government guidelines.

### Worm strains and their maintenance

The N2 strain of *C. elegans* was obtained from the *Caenorhabditis* Genetics Center (CGC, University of Minnesota, USA) and maintained using standard methods [43]. *C. elegans* mutant strain CY246 [*age-1* (*mg44*); *sqt-1* (*sc13*)/*mnC1 dpy-10* (*e128*); *unc-52* (*e444*) II], was maintained by picking wild-type phenotypes. The *age-1*^*-/-*^ F1 progeny of this strain, which are marked in *cis* with the *sqt-1* roller phenotype and exhibit maternal rescue, produce F2 progeny that constitutively form roller dauers [30]. The Haecon-5 strain of *H. contortus* was maintained in goats (raised helminth-free), which were infected intra-ruminally with 8,000 L3. Eggs, L1s, L2s and L3s were harvested from the faeces from infected goats, as described previously [40]. For the collection of L4s and adults, infected goats were euthanized with an overdose of pentobarbitone sodium (Lethobarb, Virbac) at 8 and 30 days of infection, respectively. These two developmental stages were collected from abomasa at necropsy, washed extensively in physiological saline to remove debris, and male and female worms separated prior to snap freezing in liquid nitrogen and subsequent storage at -80°C.

### DNA and RNA preparation

Genomic DNA was extracted from mixed-stage *C. elegans* (N2 strain) using the EasyPure Genomic DNA Kit (TransGen Biotech, China); total RNA was isolated using the TRIzol Plus Purification kit (Life Technologies, USA). Genomic DNA of *H. contortus* was extracted from cultured L3s, and total RNA was isolated from various stages, including eggs, first-stage larvae (L1), second-stage larvae (L2), infective L3s (iL3s), females and males of fourth-stage (L4) and adults using the same kits. RNA integrity and yields were verified by electrophoresis and spectrophotometric analysis (NanoDrop Technologies), respectively. RNA was treated with RQ1-RNase-Free DNase (Promega, USA). Nucleic acids were frozen and stored at -80°C.

### Isolation of genes and cDNAs

Guided by transcriptomic and genomic datasets for *H. contortus* [32,33], we isolated full-length *Hc-age-1* and *Hc-aap-1* cDNAs and genes. Coding regions were obtained by 5’- and 3’-rapid amplification of cDNA ends (RACE) (SMARTer RACE cDNA Amplification Kit, Clontech, USA) and cloned separately into the plasmid vector pMD-19 T (Takara, Japan) and sequenced using the primer pairs Hc-age-1 F/Hc-age-1R or Hc-aap-1 F/Hc-aap-1R (Additional file 1). Gene sequences were available from contigs [33], and exon-intron junctions were identified using the program Clustal Omega (http://www.ebi.ac.uk/Tools/msa/clustalo/). Genomic sequences upstream of the genes *Hc-age-1* and *Hc-aap-1* were sequenced as described previously [40].

### Bioinformatic analyses

Nucleotide sequences were assembled using the program CAP3 (http://seq.cs.iastate.edu/cap3.html) and compared with those in non-redundant databases using the BLAST v.2.0 suite of programs from the National Center for Biotechnology Information (NCBI; http://www.ncbi.nlm.nih.gov/BLAST), the Wellcome Sanger Centre (www.sanger.ac.uk) and the Parasite Genome (www.ebi.ac.uk) databases to verify their identity. The conceptual translation of individual cDNAs into amino acid sequences was performed using the selection “translate”, available at http://bioinformatics.org/. Protein motifs were identified by scanning the databases Pfam (www.sanger.ac.uk/Software/Pfam) and Motif Scan (http://myhits.isb-sib.ch/cgi-bin/motif_scan). Signal sequences were predicted using SignalP v.2.0 [44] available *via* the Center for Biological Sequence Analysis (www.cbs.dtu.dk/services/SignalP). Amino acid sequence alignments were carried out using the program Clustal W [45] and adjusted manually. Promoter elements in the 5’-untranslated region (UTR) were predicted using the transcription element search system available at http://www.dna.affrc.go.jp/PLACE/signalscan.html.

Amino acid sequences of *Hc*-AGE-1 and *Hc*-AAP-1 (determined in this study) and homologous sequences from other invertebrates (nematodes, *Drosophila melanogaster* and *Saccharomyces cerevisiae*) and vertebrates (human and mouse) were used for phylogenetic analyses. Sequences were aligned (as described above), and analyses conducted using the neighbor joining (NJ), maximum parsimony (MP) and maximum likelihood (ML) methods, based on the Jones-Taylor-Thornton (JTT) model [46]. Confidence limits were assessed using 1000 pseudo-replicates for NJ, MP and ML trees, and other settings were obtained using the default values using the program MEGA v.5.0 [46]. A 50% cut-off value was implemented for the consensus tree.

### Transcript abundance based on RNA-seq analysis

Transcriptomic data for different developmental stages of *H. contortus* (Haecon 5 strain, Australia), including eggs, L1s, L2s, L3s, L4 males, L4 females, adult males and adult females, were generated previously by RNA-seq [33] and made publicly available. The abundances of relevant transcripts were established as described previously [33].

### Construction of plasmids

Two constructs *Ce-age-1p*::*Ce-age-1* (102 bp)::*gfp*::*Ce-age-1 t* and *Hc-age-1p*::*Hc-age-1* (45 bp)::*gfp*::*Ce-age-1 t* (designated pL-Cagep and pL-Hagep; Additional file 2) were made. Briefly, the 1,480 bp region 5’ to *Ce-age-1* and 1,900 bp region 5’ to *Hc-age-1* were each PCR-amplified from *C. elegans* and *H. contortus* genomic DNAs, respectively. The *gfp*-coding region, with introns, was amplified from the pPD95.75 vector, and the *Ce-age-1 t* sequence was amplified from *C. elegans* genomic DNA. Then, the different elements were fused by overlap-extension PCR [30,47] employing primer pairs Ce-age-gfp-F/Ce-age-gfp-R or Hc-age-gfp-5F/Hc-age-gfp-5R (Additional file 1). The PCR products were cloned into the pGEM-T-Easy vector (Promega) and sequenced.

Using a similar strategy, two plasmid constructs *Ce-aap-1p 1515 bp*::*Ce-aap-1* (24 bp)::*gfp*::*Ce-unc-54 t* and *Hc-aap-1p 2524 bp*::*Hc-aap-1* (36 bp)::*gfp*::*Ce-unc-54 t* (designated pL-Caapp and pL-Haapp) containing the promoters, the *gfp* reporter and *Ce-unc-54 t* (terminator) were made. In brief, the 1,515 bp region 5’ to *Ce-aap-1* and 2,524 bp region 5’ to *Hc-aap-1* were PCR-amplified from *C. elegans* and *H. contortus* genomic DNAs, respectively. The *gfp* and *Ce-unc-54 t* fragments were amplified from the pPD95.75 vector. Then, the promoter, *gfp* and terminator were fused by overlap-extension PCR using primer pair Ce-aap-gfp-F/Ce-aap-gfp-R or Hc-aap-gfp-F/Hc-aap-gfp-R (Additional file 1). Finally, the amplicons were cloned into pGEM-T-Easy and sequenced.

For the gene rescue assay, the constructs *Ce-age-1p*::*Ce-age-1* (3,549 bp)::*Ce-unc-54 t* (designated pL-Cage1), *Ce-age-1p*::*Hc-age-1* (3,471 bp)::*Ce-unc-54 t* (designated pL-Hage1) and *Ce-aap-1p*::*Hc-aap-1* (1,284 bp)::*Ce-unc-54 t* (designated pL-Haap1) were made (Additional file 2). The inferred promoters of *Ce-age-1* (1,275 bp) and *Ce-aap-1* (1,515 bp) were amplified from genomic DNA of *C. elegans* and sequenced; *Ce-unc-54 t* was from vector pPV238; the coding sequences of *Ce-age-1*, *Hc-age-1* & *Hc-aap-1* were amplified from *C. elegans* and *H. contortus* cDNAs, respectively. Then, three fragments, including the *Ce-age-1* promoter, *Ce-age-1* coding sequence and *Ce-unc-54 t* were fused to create pL-Cage1 by overlap-extension PCR. The pL-Hage1 plasmid was made from the *C. elegans* vector pPV238. Briefly, pPV238 was cut at the *Pst*I and *Bst*Z17I restriction sites, and the 5’ region of *Ce-age-1* added, with the *Mlu*1 restriction site upstream of *Bst*Z171 being inserted. Then, the *Hc-age-1* coding region with *Mlu*I and *Bst*Z17I was inserted to create pL-Hage1 using the primer pairs Ce-age-pstF/Ce-age-mlbstR and Hc-age-mluF/Hc-age-bstzR (Additional file 1). Similarly, vector pPV199 was cut at the endonuclease sites *Bam*HI, *Age*I or *Mlu*I, and the *Ce-aap-1* promoter and *Hc-aap-1* coding regions were inserted, in turn, to create the pL-Haap1 using the PCR primer pairs Ce-aap-pbamF/Ce-aap-pageR and Hc-aap-ageF/Hc-aap-mluR, respectively (Additional file 1).

### Yeast two-hybrid assay

A yeast two-hybrid system (Gal4-based Matchmaker Gold, Clontech) was used to test interactions between the adaptor-binding domain of *Hc*-AGE-1 and *Hc*-AAP-1 [48,49]. Translational fusion to the Gal4-activation domain (AD) was conducted as follows: a full-length *Hc-aap-1* cDNA was PCR-amplified from *H. contortus* using the primer pair Hc-aap-NdeF/Hc-aap-BamR (Additional file 1) and cloned into the vector pGADT7 *via* the *Nde*I and *Bam*HI restriction sites. To construct a translational fusion to the Gal4-binding domain (BD), a cDNA fragment encoding the adaptor-binding domain of *Hc-age-1* PCR-amplified from *H. contortus* using primer pair Hc-age-NdeF/Hc-age-BamR was cloned into the vector pGBKT7 *via Nde*I and *Bam*HI sites.

The constructs fused to *Hc-aap-1* or *Hc-age-1* were sequenced to verify their reading frames and then transformed into yeast strains Y2HGold and Y187. Interaction trap analysis of diploids, resulting from the mating of Y2HGold and Y187 strains, was carried out using the manufacturer’s protocol (Clontech) employing minimal medium agar plates lacking leucine, tryptophan (plasmid selection marker) and either histidine (low stringency for protein-protein interaction), histidine (in the presence of 1.5 μM 3-amino-triazole; medium stringency) or histidine plus adenine (high stringency). Colonies were assessed after 5 days of incubation at 30°C. The yeast-two hybrid assay was repeated twice.

### Transformation of *C. elegans*

The N2 or CY246 (wild-type) strain of adult hermaphrodites of *C. elegans* were transformed using a gonad microinjection method [50]. In brief, to study the expression patterns of *Ce-age-1*, *Hc-age-1*, *Hc-aap-1* and *Ce-aap-1* promoters, each of the constructs was microinjected with 20 ng/μl for pL-Cagep, pL-Hagep, pL-Caapp or pL-Haapp, together with 80 ng/μl PRF4 containing the *rol-6* marker gene. Injected worms were transferred to nematode growth medium (NGM) plates with *Escherichia coli* (OP50) lawns and incubated at 20°C. Transformed worms were identified among F1 progeny based on a “right-roller” and the green fluorescence protein (GFP) phenotype. Then, transformants were anaesthetized using 10 mM levamisole and immobilized on a 2% agar pad, and examined using a stereoscopic and compound fluorescence microscope, equipped with differential interference contrast optics (DIC) and a camera (Olympus BX51, Japan).

### Gene rescue assay in the mutant strain CY246 of *C. elegans*

To assess the ability of *Hc-age-1* to rescue *age-1* in *C. elegans* (mutant strain CY246), three plasmids (pL-Cage1, pL-Hage1 and pL-Haap1; Additional file 2) were constructed. Each of the plasmids was injected with 20 ng/μl of test plasmid and co-injected with 2 ng/μl of the marker *Ce-myo-2* p::*mCherry*::*Ce-unc-54 t* (pCFJ90) and 78 ng/μl of pUC19. Additionally, worms were injected with the same amount of the marker pCFJ90 and 98 ng/μl of pUC19 alone, as controls. Injected worms were transferred to NGM + OP50 plates, and transformants identified by fluorescence microscopy. Lines were established from single F2 transformants. The pL-Cage1 and pL-Hage1 lines were assessed for gene rescue based on the presence of progeny from F1 rollers and the ability of F2 rollers to passage for ≥10 generations.

## Results

### Characterization of cDNAs and phylogenetic analysis of amino acid sequence data

**Figure 1:**
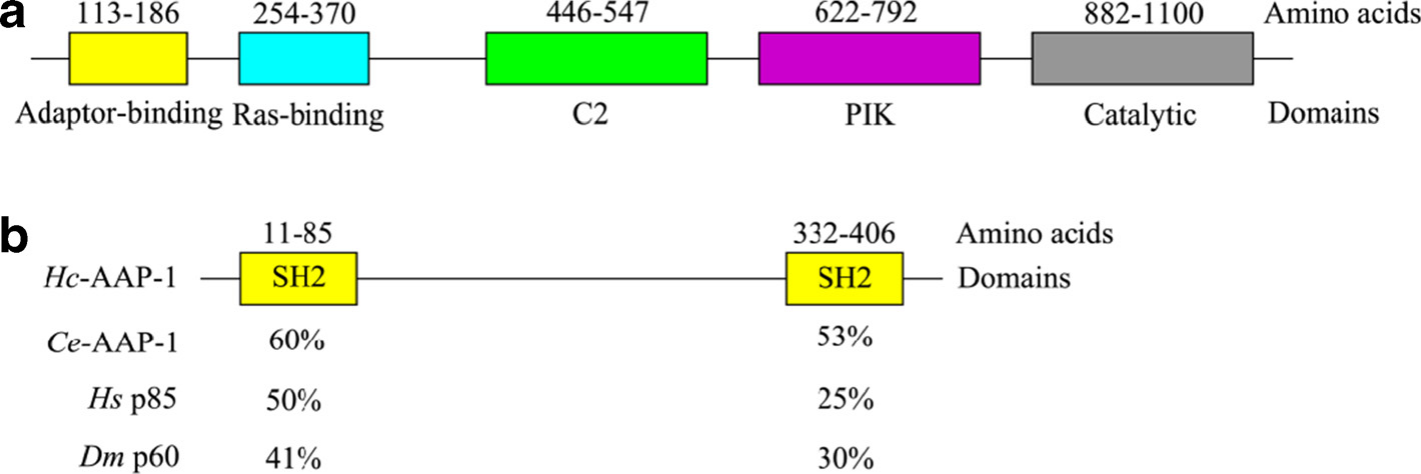
**Main functional domains of phosphoinositide 3-kinase (PI3K) catalytic subunit** ***Hc*** **-AGE-1 and the regulatory subunit** ***Hc*** **-AAP-1 of** ***Haemonchus contortus*** **. (a)** Colours represent different functional domains: adaptor-binding domain (yellow), Ras-binding domain (light blue), C2 domain (green), PIK domain (purple) and the catalytic domain (grey). **(b)** N-terminal and C-terminal SH2 domains (yellow). Numbers represent amino acid positions. Percentage under the amino and carboxylic terminal SH2 domain shows the similarity to the SH2 domains of homologues from various eukaryotes, including *Caenorhabditis elegans*, *Drosophila melanogaster* and *Homo sapiens*.

The *Hc-age-1* cDNA was 3,471 bp in length and encoded a protein (*Hc-*AGE-1) of 1,156 amino acids. *Hc*-AGE-1 had 31-44% amino acid sequence similarity to homologues from *Ascaris suum*, *C. elegans*, *Loa loa*, *Parastrongyloides trichosuri* and *S. stercoralis* as well as *Homo sapiens* and *Mus musculus. Hc*-AGE-1 consists of five conserved domains, including an adaptor-binding domain proposed to interact with a regulatory subunit (a Ras-binding domain that mediates activation by the small GTPase Ras) and the C2-like, phosphatidylinositol kinase and catalytic domains (Figure 1a). By contrast, the *Hc-aap-1* cDNA was 1,284 bp in length and encoded a protein (*Hc-*AAP-1) of 427 amino acids. *Hc*-AAP-1 had 29-40% similarity to homologues from *Brugia malayi*, *C. brenneri*, *C. elegans* and *L. loa*. The *Hc*-AAP-1 sequence included N-terminal SH2 (Src-homolog 2), C-terminal SH2 domains and an inter-SH2 domain required for binding to the catalytic subunit [51] (Figure 1b).

**Figure 2:**
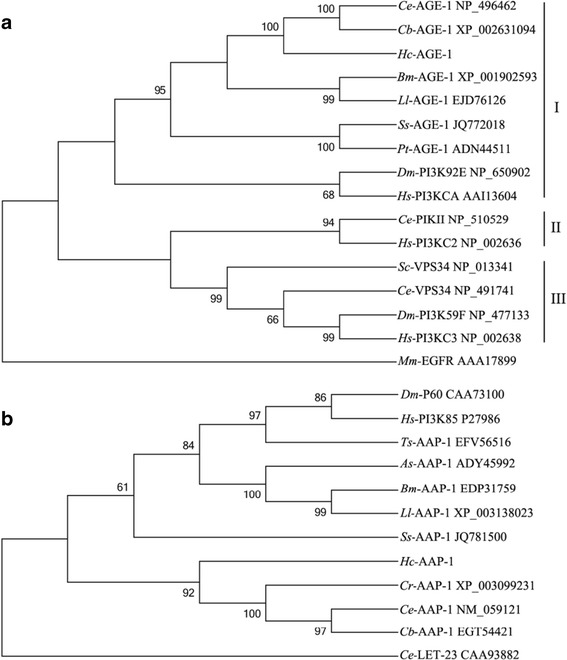
**Neighbor joining trees showing the relationship of** ***Haemonchus contortus*** **phosphoinositide 3-kinase (PI3K) catalytic subunit** ***Hc*** **-AGE-1 and regulatory subunit** ***Hc*** **-AAP-1.** The trees were calculated using the Jones-Taylor-Thornton (JTT) model in the MEGA program version 5.0. Bootstrap values above or below the branches (1,000 iterations) are shown for robust clades (>50 %). **(a)** The species used in the analysis include six nematodes (*Strongyloides stercoralis*, *Ss*-AGE-1; *Parastrongyloides trichosuri*, *Pt*-AGE-1; *Brugia malayi*, *Bm*-AGE-1; *Loa loa*, *Ll*-AGE-1; *Caenorhabditis elegans*, *Ce*-AGE-1, *Ce*-PIKII and *Ce*-VPS34; *C. briggsae*, *Cb*-AGE-1) and three non-nematodes (*Drosophila melanogaster*, *Dm*-PI3K92E and *Dm*-PI3K59F; *Homo sapiens*, *Hs*-PI3KCA, *Hs*-PI3KC2 and *Hs*-PI3KC3; *Saccharomyces cerevisiae*, *Sc*-VPS34). *Mus musculus* EGFR AAA17899 was used as an outgroup. **(b)** AAP-1 used in the analysis included *Caenorhabditis elegans*, *Ce*-AAP-1; *C. briggsae*, *Cb*-AAP-1; *C. remani*, *Cr*-AAP-1; *Strongyloides stercoralis*, *Ss*-AAP-1; *Ascaris sum*, *As*-AAP-1; *Brugia malayi*, *Bm*-AAP-1; *Loa loa*, *Ll*-AAP-1; *Trichinella spiralis*, *Ts*-AAP-1; *Drosophila melanogaster*, *Dm*-P60; *Homo sapiens*, *Hs*-PI3K85). *C. elegans Ce*-LET-23 CAA93882 was used as an outgroup. GenBank accession numbers are listed on the right of each species.

The *Hc*-AGE-1 sequence was aligned with 14 AGE-1 homologues (from six nematodes and three eukaryotes) and subjected to phylogenetic analyses (Figure 2a). There was concordance in topology among the MP, ML and NJ trees, which all displayed three strongly supported clusters representing classes I, II and III, respectively. *Hc*-AGE-1 had the closest relationship to class I homologs from *C. elegans* and *C. briggsae.* The predicted amino acid sequence of *Hc*-AAP-1 was aligned with ten AAP-1 homologues (from eight nematodes and two other metazoans) and subjected to phylogenetic analyses (Figure 2b). Again, there was concordance in topology among the NJ, MP and ML trees which showed that *Hc*-AAP-1 has its closest relationship with *Caenorhabditis* homologs, to the exclusion of AAP-1 s from *S. stercoralis* as well as *A. suum*, *B. malayi* and *L. loa*, with the AAP-1 s from the latter three species grouping together with absolute nodal support (100%). Interestingly, AAP-1 of *Trichinella spiralis* grouped with *D. melanogaster* and *H. sapiens* homologues, with 97% support (Figure 2b).

### Genomic organization and putative promoter elements

**Figure 3:**
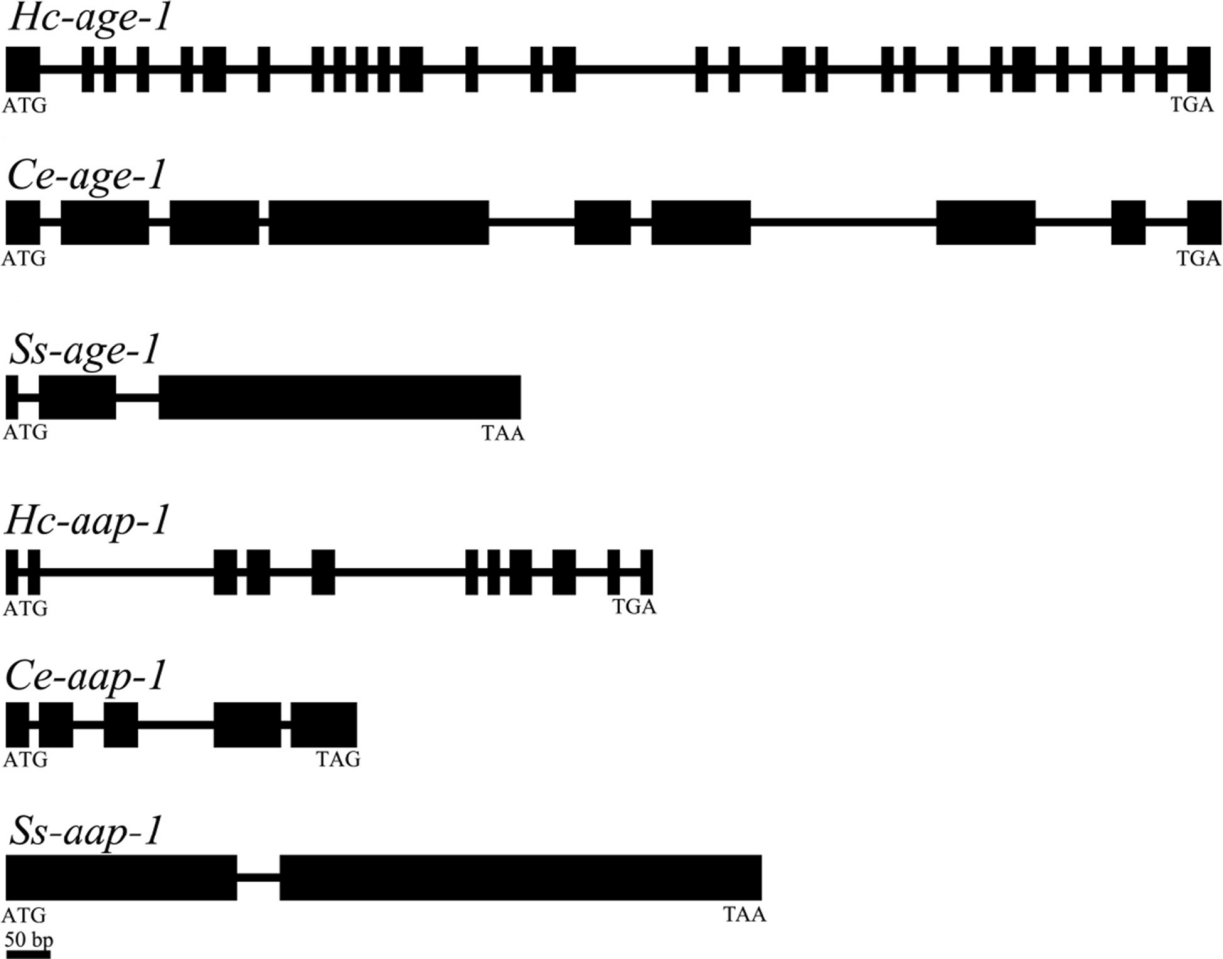
**Schematic diagram displaying the genomic organization of** ***age-1*** **and** ***aap-1*** **from** ***Haemonchus contortus*** **(** ***Hc-age-1*** **and** ***Hc-aap-1*** **),** ***Caenorhabditis elegans*** **(** ***Ce-age-1*** **and** ***Ce-aap-1*** **) and** ***Strongyloides stercoralis*** **(** ***Ss-age-1*** **and** ***Ss-aap-1*** **).** Black boxes represent exons. Lines between the exons represent introns. Start (ATG) or stop (TAG/TGA/TAA) codons are indicated.

We located the full-length genes of *Hc-age-1* and *Hc-aap-1* in the *H. contortus* genome [33]. The *Hc-age-1* gene was 12,896 bp in length and had 29 exons (75-198 bp); the *Hc-aap-1* gene was 6,172 bp in length and contained 11 exons (69-179 bp) (Figure 3). All exon/intron boundaries abided by the GT-AG rule in *Hc-aap-1* (Additional file 3), with the exception of the first and fifth exons in *Hc-age-1* (Additional file 4). *Hc-age-1* and *Hc-aap-1* had more exons and introns compared with orthologous genes in *C. elegans* [12,13] and *S. stercoralis* [30]. The promoter regions predicted for *Hc-age-1* and *Hc-aap-1* were 1,900 bp and 2,524 bp in length, respectively. In the promoter regions of both genes, E- (CANNTG), inverse GATA (TTATC), inverse CAAT (ATTGG), CAAT (CCAAT) and TATA boxes were identified.

### Transcriptional profiles in *H. contortus*, and localization of expression in transgenic *C. elegans*

**Figure 4:**
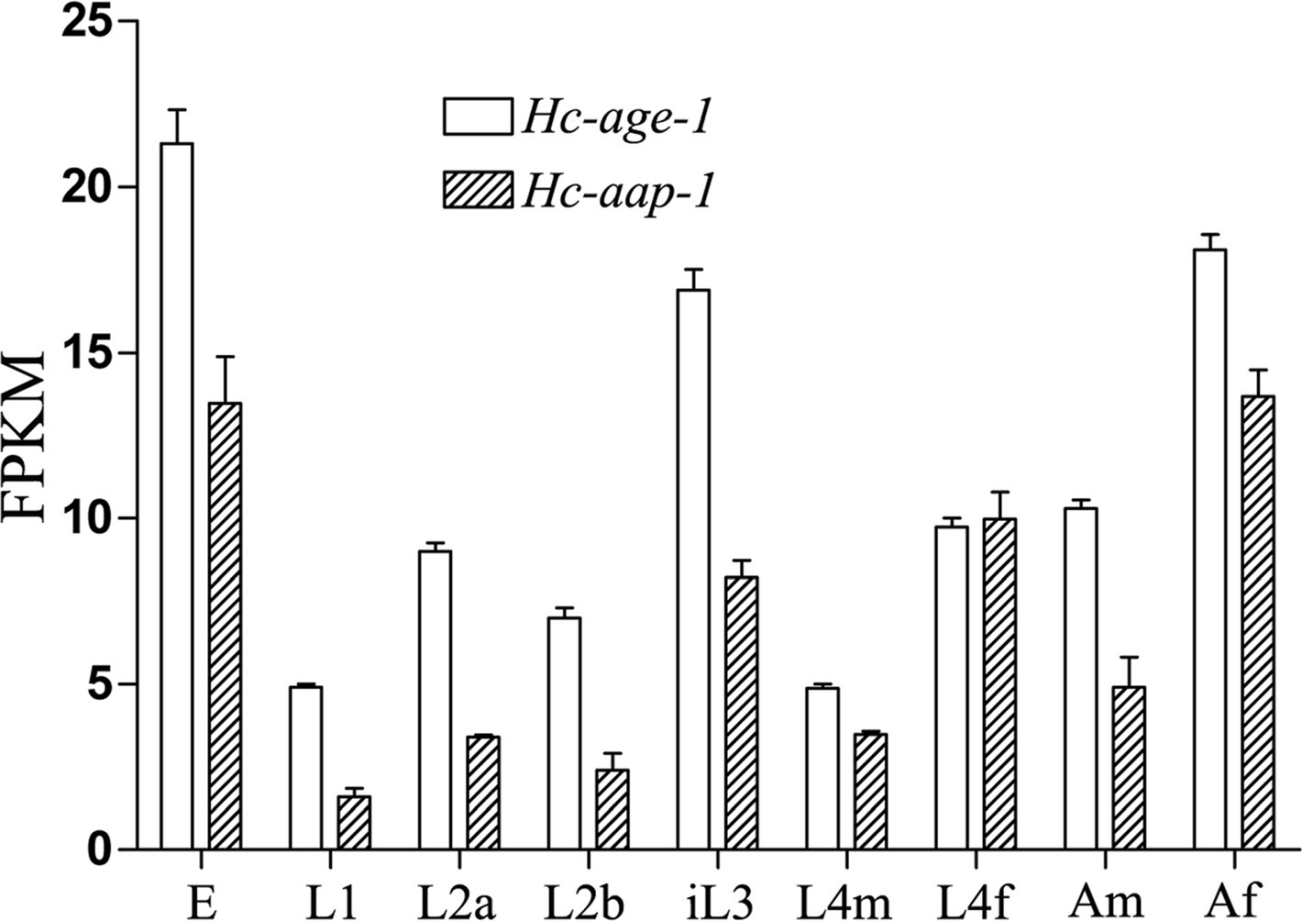
**Developmental profile of transcriptional abundance for** ***Hc-age-1*** **(white bar) and** ***Hc-aap-1*** **(striped bar) in different developmental stages of** ***Haemonchus contortus*** **.** Transcript abundance were compared among eight developmental stages, each in biological triplicate (n =3); eggs (E), the first-stage larvae (L1), the second-stage larvae (L2), the infective L3 (iL3), the fourth-stage males (L4m), the fourth-stage females (L4f), adult males (Am) and adult females (Af). Transcript abundances were counted as fragments per kilobase of coding exon per million mapped reads (FPKM). Error bars represent 95 % confidence intervals.

**Figure 5:**
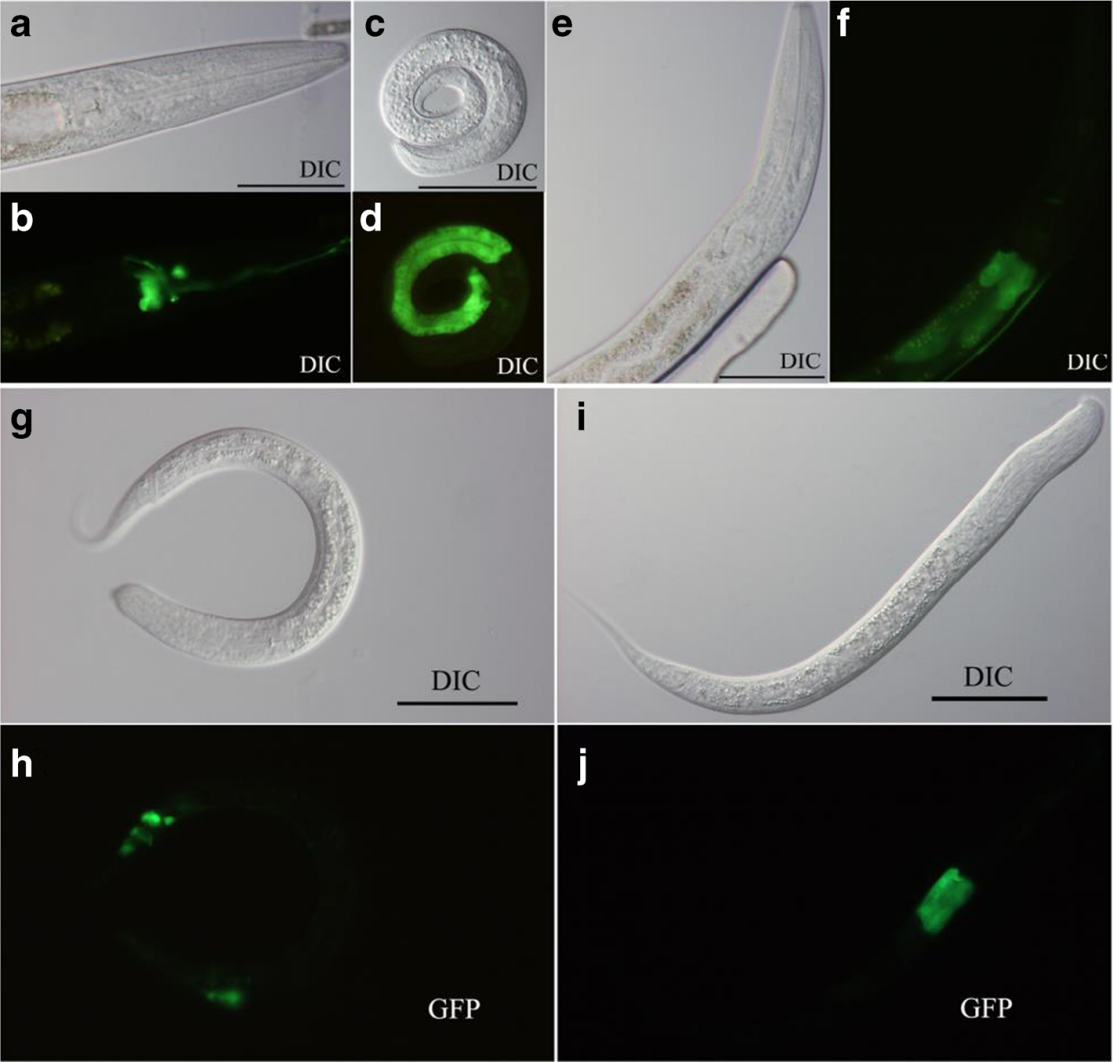
**Expression patterns of** ***Hc-age-1*** **and** ***Hc-aap-1*** **.** Representative expression patterns of *Ce-age-1*, *Hc-age-1*, *Ce-aap-1* and *Hc-aap-1* displayed in *Caenorhabditis elegans* transformed by either of the four green fluorescent protein (GFP) constructs pL-Cagep, pL-Hagep, pL-Caapp and pL-Haapp (Additional file 2). Three transgenic lines were produced for each of the four plasmids. **(a and b)** Differential interference contrast (DIC) **(a)** and fluorescence **(b)** images of transgenic third-stage larva (L3) of *C. elegans* expressing *Ce-age-1*p::*gfp* (pL-Cagep). **(c and d)** DIC **(c)** and fluorescence **(d)** images showing the expression pattern of *Hc-age-1* p::*gfp* (pL-Hagep) in first-stage larva (L1). **(e and f)** DIC **(e)** and fluorescence **(f)** images, showing the expression pattern of *Hc-age-1*p::*gfp* (pL-Hagep) in L3. **(g and h)** DIC **(g)** and fluorescence **(h)** images showing expression pattern of *Ce-aap-1* p::*gfp* (pL-Caapp) in L3. (I and J) DIC **(i)** and fluorescence **(j)** images showing expression pattern of *Hc-aap-1* p::*gfp* (pL-Haapp) in L3. Scale bars =50 μm.

*Hc-age-1* and *Hc-aap-1* were transcribed in all developmental stages of *H. contortus*. Highest transcription was measured in eggs, L3s and female adults (*Hc-age-*1), or eggs, L3s, female L4s and female adults (*Hc-aap-1*) (Figure 4). Expression was localized in *C. elegans* (N2) transformed with plasmid pL-Hagep containing the 1,900 bp-predicted promoter region of *Hc-age-1* or plasmid pL-Haapp containing the 2,524 bp-predicted promoter region of *Hc-aap-1*, using pRF4 as a marker. In addition, *C. elegans* (N2) transformed with *gfp* fused to each *Ce-age-1* and *Ce-aap-1* constructs were used as controls. The transgenic lines were screened for the roller phenotype and GFP expression. GFP expression driven by the *Ce-age-1* promoter was detected in amphidial neurons and the intestine, consistent with previous reports for *S. stercoralis* [30]. GFP expression driven by the *Hc-age-1* promoter was detected in the entire intestine at L1 and L2 stages, but concentrated in the anterior intestine of the L3 stage (Figure 5c-f). Similarly, GFP expression driven by the *Ce-aap-1* promoter was observed in intestine and amphidial/phasmidial neurons (Figure 5g-h) in accordance with previous report [13]. The expression of *Hc-aap-1* was also predominantly present in anterior intestine, which is highly analogous to that of *Hc-age-1* (Figure 5i-j). Despite some variation in expression among individual larvae, *C. elegans* containing *Ce-age-1* p::*gfp* and *Ce-aap-1* p::*gfp* showed a similar expression profile (amphidial/head neurons and intestine), as did *C. elegans* containing *Hc-age-1* p::*gfp* and *Hc-aap-1* p::*gfp* (anterior intestine). Based on their apparent co-location, *Hc*-AGE-1 and *Hc*-AAP-1 might interact as a heterodimer [52].

### Assessing the interaction of AAP-1 with AGE-1

In multicellular organisms, binding by the adaptor subunit (*Ce*-AAP-1) can stabilize and activate the PI3K catalytic subunit (*Ce*-AGE-1) [11,13,51]. The interaction of these two subunits is governed by the NH2 terminus of the catalytic subunit and the inter-SH2 domain of the adaptor subunit [51]. To assess whether there was an interaction between *Hc*-AAP-1 and the adaptor-binding domain of *Hc*-AGE-1, several fusion constructs in the vectors pGADT7 and pGBKT7, respectively, were prepared for analysis in the yeast two-hybrid system. Co-expression of the full-length gene *Hc-aap-1* fused to pGADT7 (GAL4 AD-*Hc*-AAP-1) with the adaptor-binding domain of *Hc*-*age-1* fused to pGBKT7 (GAL4 BD-*Hc*-AGE-1) resulted in high β-galactosidase expression activity (Additional file 5). The co-expression of pGADT7 or pGBKT7 fused to the large T-antigen or murine p53 (pGADT7-T and pGBKT7-53, respectively; positive controls) displayed β-galactosidase expression (Additional file 5). In contrast, the co-expression of pGADT7-*Hc*-*aap-1* with pGBKT7-53 or pGBKT7-*Hc*-*age-1* with pGADT7-T did not show such expression (Additional file 5). These findings indicated that the adaptor-binding domain of *Hc*-AGE-1 was capable of interacting with *Hc*-AAP-1 in the yeast two-hybrid system.

### Attempts of cross-species complementation in *C. elegans* CY246 with *Hc-age-1* and *Hc-aap-1*

*C. elegans* mutant strain CY246 [*age-1* (mg44) mutant] could be fully rescued by the homologous gene in pL-Cage1 (Additional file 2). Rescue was evidenced based on the presence of F1 roller progeny, and F2 rollers were passaged for ≥10 generations. This result suggests that the main regulatory and coding regions are functional in *age-1* (mg44) mutants, thus providing a reliable basis for comparative studies of *Hc*-AGE-1.

To attempt to establish the functional characteristics of the *Hc-age-1* gene, the construct comprising the *Ce-age-1* promoter and the *Hc-age-1* coding region (pL-Hage1, Additional file 2) was transformed into the CY246 mutant strain. *Hc*-*age-1* mRNA was detected in all lines of the CY246 strain transformed with the rescuing construct. Three lines were screened, and gene rescue was attempted. However, no individuals from the progeny of *age-1*^-/-^*C. elegans* transformed with pL-Hage1 were rescued.

Although the *Ce-age-1* promoter was functional in the positive control, *Hc-age-1* did not rescue the mutant strain. A possible explanation is that the heterodimer was processed incorrectly, either by the thermally labile AGE-1 subunit or by the inhibition of catalytic activity by a conformational disorder. To address this proposal, the plasmid (pL-Haap1) containing the *Hc-aap-1* coding sequence, under the control of the *Ce-aap-1* promoter, was constructed (Additional file 2). Although *Hc*-AAP-1 conferred a strong interaction with the *Hc*-AGE-1 catalytic subunit in a yeast-two hybrid assay, the transgenic lines transformed with both the pL-Haap1 and pL-Hage1 constructs did not rescue *C. elegans age-1* (mg44) mutants.

## Discussion

In *C. elegans*, signalling through the phosphoinositide 3-kinases heterodimer is a key regulator of the switch from dauer to continuous development [5,13]. PI3Ks, which are relatively conserved enzymes from unicellular eukaryotes to multicellular organisms, play important roles in intracellular trafficking, cell metabolism, differentiation and/or survival [53-56]. Recently, components of insulin-like signalling have been identified in parasitic nematodes, including *A. caninum* [37,38], *H. contortus* [39,40] and *S. stercoralis* [30,41,42,57], suggesting that similar signalling is utilized by both free-living and parasitic nematodes. In the present study, two PI3K genes were identified in *H. contortus* using available data [33], including a *Hc-age-1* encoding a p110 catalytic subunit and a *Hc-aap-1* encoding a p50/p55-like regulatory subunit, consistent with those in *C. elegans* and *S. stercoralis* [12,13,30].

Sequence and structural analyses revealed that *Hc*-AGE-1 and *Hc*-AAP-1 are relatively conserved among *H. contortus*, *C. elegans* and other nematodes. *Hc*-AGE-1 has representative structural domains, including an N-terminal adaptor-binding domain, inferred to interact with the adaptor regulatory subunit, a Ras-binding domain predicted to regulate activation by the small GTPase Ras, a C2 domain, a phosphatidylinositol kinase homology (PIK) domain and a C-terminal catalytic domain (cf. Figure 1). *Hc*-AAP-1 has a common structural domain possessing an inter-SH2 domain, flanked by both Src-homology 2 domains (SH2) and necessary for binding to the catalytic subunit (cf. Figure 1). In bovine PI3K, although the inter-SH2 of the regulatory subunit confers a strong interaction with the catalytic subunit, more than one SH2 domain, in combination with inter-SH2 domain, are required for the conformational changes required to activate the p110 subunit through phosphotyrosine peptide binding [51]. Hence, it was reasonable to hypothesize that the *Hc*-AAP-1 regulatory subunit can bind the *Hc*-AGE-1 catalytic subunit to assemble the PI3K heterodimer. This hypothesis was tested using the yeast two-hybrid assay, revealing that the adaptor-binding domain of *Hc*-AGE-1 readily and strongly interacts with the *Hc*-AAP-1 regulatory subunit, analogous to orthologous molecules in *C. elegans* and *D. melanogaster* [13,58]. Phylogenetic analysis of inferred amino acid sequences showed that *Hc*-AGE-1 and *Hc*-AAP-1 grouped with the orthologues *Ce*-AGE-1 and *Cb*-AGE-1 and *Cr*-AAP-1 (free-living nematodes), respectively. In addition, AGE-1 and AAP-1 orthologues are present in various parasitic nematodes, including *A. suum*, *B. malayi* and *S. stercoralis*. Therefore, these findings suggest that the *Hc*-AGE-1/*Hc*-AAP-1PI3 heteromer kinase is generally conserved and might, thus, have similar functional characteristics in various nematodes.

Some genes are transcriptionally regulated during the key transition associated with the substantial growth and development in nematodes, such as the switch from the free-living to parasitic stages for parasitic nematodes and the transition from the arrested to the developmentally active state in *C. elegans* [30,33]. Overall, an assessment of transcription showed that *Hc-age-1* and *Hc-aap-1* were transcribed at a low level throughout the life cycle of *H. contortus*, like *Ce-age-1* and *Ss-age-1*, which is in accordance with the hypothesis that the insulin-like signalling is regulated post translationally by AGE-1, rather than at the transcriptional level. Furthermore, the *Hc-age-1* and *Hc-aap-1* were transcriptionally up-regulated in eggs, infective L3s and female adults of *H. contortus*, which suggests the PI3K might be involved in nematode development and/or reproduction. Significantly, up-regulation in iL3, which is akin to a developmentally arrested stage, and has a significantly reduced metabolic rate and transcription [33], hints to PI3K having a functional role in recovery from developmental arrest.

The expression of *Hc-age-1* was mainly localised to the intestine in the larval stages of *C. elegans*, which is consistent with the expression pattern of the *Ce-age-1*, gleaned from high throughput analyses in *C. elegans* (see WormAtlas: http://gfpweb.aecom.yu.edu/strain?name=BC10837), where the *Ce-age-1* promoter::*gfp*-fusion construct is expressed in the intestine and amphidial/head neurons. Similarly, the *Hc-aap-1* promoter::*gfp*-fusion construct was concentrated in the anterior intestine, which is in accord with the *Ce-aap-1* promoter [13], where the expression of *Ce-aap-1* promoter::*gfp*-fusion plasmid was in intestine and head/tail neurons. The expression of *Hc-age-1* and *Hc-aap-1* showed a strong spatio-temporal relationship with a promoter-promoter interaction [52], which may indicate a functional correlation. Indeed, *Hc*-AGE-1 and *Hc*-AAP-1 can bind tightly to form a PI3K heterodimer, as confirmed by yeast two-hybrid analysis and as predicted also for a multicellular organism, including bovine, *C. elegans* and mouse [13,51,59]. To some extent, the heterologous expression pattern might enable us to mine for protein-protein interaction information by determining in which tissues or cells these proteins are co-expressed. In comparison with *C. elegans*, the expression of *Hc-age-1* and *Hc-aap-1* was mainly concentrated in the intestine rather than the nervous systems, which might suggest that PI3K plays a crucial role in feeding in parasitic nematodes. Thus, like in *A. caninum*, *A. ceylanicum* and *S. stercoralis*, the PI3K specific inhibitor LY294002 inhibits the resumption of feeding [30,31].

Based on our findings, we suggest that *Hc-age-1* is a functional orthologue of *Ce-age-1*, and could be activated by upstream growth factor receptor tyrosine kinases (RTKs), and negatively regulates the Foxo-class transcription factor DAF-16. To test this proposal, heterologous genetic complementation was applied in *C. elegans*. The *C. elegans* mutant strain CY246 was fully rescued by the *Ce-age-1* construct, indicating that the *Ce-age-1* promoter can direct suitable expression and appropriate heterodimer binding to enable signalling. However, rescue was not achieved when the *Hc-age-1* construct was introduced into the CY246 strain. A possible explanation is that the exogenous *Hc*-AGE-1 subunit cannot bind the endogenous *Ce*-AAP-1 subunit *in vivo* to constitute the functional PI3K. It is proposed that *Hc*-AGE-1 and *Hc*-AAP-1 bind to each other to constitute a functional PI3K in transgenic *C. elegans*, rather than a single thermally labile AGE-1 subunit [59]. However, we were unable to detect any transgenic lines (transformed with two plasmids containing *Hc-age-1* and *Hc-aap-1* together) that could be passaged.

In strongylid nematodes, the resumption of feeding has been used as a phenotypic marker in *in vitro*-activation assays [30,31,60,61], and the PI3 kinase-specific inhibitor LY294002 effectively blocks the resumption of feeding of exsheathed L3s [30,31], which infers that PI3K plays a key role in the activation of L3s of parasitic nematodes, as it does the exit from dauer in *C. elegans*. Regarding the failure of *Hc-age-1* to complement the *C. elegans age-1* (mg44) mutant strain, a possible explanation is that the proposed orthologues of *Hc-age-1* and *Hc-aap-1* are unable to assemble the functional heterodimer and/or attain their native conformation, because of a lack of molecular chaperones, such as calnexin and calreticulin [62,63], even though they bind tightly to each other in the yeast two-hybrid system. Additionally, the evolution of gene regulation might relate to variation in the temporal and spatial control of gene expression pattern in tissues [64]. We observed that the expression patterns of *Hc-age-1* and *Hc-aap-1* tend to be in the intestine of *C. elegans* compared with those of *Ce-age-1* and *Ce-aap-1*, with expression mainly in head neurons. This difference might suggest some functional variation of PI3K between free-living and parasitic nematodes.

The “dauer hypothesis” proposes that similar molecular signalling pathways control the entry into and exit from arrested development in *C. elegans* and in parasitic nematodes [65,66]. Heterologous complementation showed that the main components of insulin-like signalling, including *daf-16, daf-2*, *age-1* and *daf-12* in parasitic nematodes [37,39-41,67], have similar functions to *C. elegans* orthologues. However, it is, nonetheless, interesting to note that the roles of PI3K from *H. contortus* appear to be divergent. Determining the lipid and protein kinase activities of PI3K in *H. contortus* might help establish whether PI3Ks or PI3K signalling is conserved or not. Some new heritable, transgenic elements or tools applied in the “model nematodes” *S. stercoralis* and *P. trichosuri*, including *piggyback* [68], might help address this and other questions relating to the insulin-like signalling pathway.

## Conclusions

In conclusion, we investigated the PI3K-encoding genes *Hc-age-1* and *Hc-aap-1* in the parasitic nematode *H. contortus*. We characterized their cDNAs, genomic DNAs and promoter sequences, and determined their transcription profiles in various developmental stages. Then, we confirmed the interaction of *Hc-age-1* and *Hc-aap-1* by yeast two-hybrid analysis. Finally, we predicted function by gene expression location and complementation in heterologous *C. elegans*. Taken together, the present results provide important insights into the characteristics of PI3K in a strongylid nematode, particularly in relation to developmental processes.

## Additional files

Additional file 1:
**Primers used to isolate** ***Hc-age-1*** **and** ***Hc-aap-1*** **genes of** ***Haemonchus contortus*** **and to make constructs for green fluorescent protein (GFP) localization in** ***Caenorhabditis elegans*** **and the heterologous genetic complementation assay.**
Additional file 2:
**Cloning strategy for reporter and rescuing constructs.** The constructs containing the *Caenorhabditis elegans Ce-age-1* and *Ce-aap-1* promoters (pL-Cagep and pL-Caapp) and the *Haemonchus contortus Hc-age-1* and *Hc-aap-1* promoters (pL-Hagep and pL-Haapp) were made based on pPD95.75 by the overlapping extension PCR. The rescuing constructs (pL-Cage1, pL-Hage1 and pL-Haap1) containing the *Ce-age-1*, *Hc-age-1* and *Hc-aap-1* coding regions, respectively, were made by removing the *gfp* coding sequence from pPV238 or pPV199 (cf. 57) and linking the appropriate cDNA. A, B, M and P represent the restriction sites for *Age*I, *Bst*Z17I, *Mlu*I and *Pst*I, respectively. Additional file 3:
**The length of exon and intron and splice donor sequences for each exon and intron of** ***Hc-aap-1*** **gene.** The dinucleotide consensus sequence at the splice site is italicized and underlined. All nucleotide sequences are 5’ to 3’. Additional file 4:
**The length of exon and intron and splice donor sequences for each exon and intron of** ***Hc-age-1*** **gene.** The dinucleotide consensus sequence at the splice site is italicized and underlined. All nucleotide sequences are 5’ to 3’. Additional file 5:
**The adaptor-binding domain of** ***Hc*** **-AGE-1 interacted with** ***Hc*** **-AAP-1 in a yeast two-hybrid assay.** (A) The positive control showed that pGBKT7-53 can interact with pGADT7-T; (B) The negative control showed that pGBKT7-Lam and pGADT7-T did not interact; (C) The test group pGBKT7-*Hc-age-1* and pGADT7-*Hc-aap-1* did interact with QDO/X/A; (D) pGBKT7-Lam and pGADT7-*Hc-aap-1* did not interact with QDO/X/A; (E) pGBKT7-*Hc-age-1* and pGADT7-T did not interact with QDO/X/A.

## Abbreviations

DIC: Differential interference contrast optics
GFP: Green fluorescence protein
JTT: Jones-Taylor-Thornton model
L3s: The third-stage larvae
ML: Maximum likelihood
MP: Maximum parsimony
NGM: Nematode growth medium
NJ: Neighbour-joining
PCR: Polymerase chain reaction
RACE: Rapid amplification of cDNA ends
UTR: Untranslated region
