# Supplementary material for: Exploring the role of two interacting phosphoinositide 3-kinases of Haemonchus contortus
Source: Parasit Vectors. 2014 Nov 12;7:498. doi: 10.1186/s13071-014-0498-2 (PMC4233088; doi:10.1186/s13071-014-0498-2)
Supplement: Additional file 1: — Primers used to isolate Hc-age-1 and Hc-aap-1 genes of Haemonchus contortus and to make constructs for green fluorescent protein (GFP) localization in Caenorhabditis elegans and the heterologous genetic complementation assay. [file 13071_2014_498_MOESM1_ESM.doc]

| **Primer Name** | **Primer Sequence (5’ to 3’)** |
| --- | --- |
| **Hc-age-1F** | ATGAGCGGTGCTGCTGTGGCCG |
| **Hc-age-1R** | TCAAATGTGCTTGACAGCGTGG |
| **Hc-aap-1F** | ATGGCTGTCGGGCAGCTCTCCGACC |
| **Hc-aap-1R** | TCATAAGGCTGGCATAGTTAGAG |
| **Ce-age-gfp-F** | ACGGACGGAACTCCCGACGTATCATGAGTAAAGGAGAAGAACTTTTC |
| **Ce-age-gfp-R** | GAAAAGTTCTTCTCCTTTACTCATGATACGTCGGGAGTTCCGTCCGT |
| **Hc-age-gfp-5F** | CCGCAAGCTCTGCCGGCGATGACCTCATGAGTAAAGGAGAAGAACTTTTC |
| **Hc-age-gfp-6R** | GAAAAGTTCTTCTCCTTTACTCATGAGGTCATCGCCGGCAGAGCTTGCGG |
| **Ce-aap-gfp-F** | ATGAGCACAACACCTGGAACTCCTATGAGTAAAGGAGAAGAACTTTTC |
| **Ce-aap-gfp-R** | GAAAAGTTCTTCTCCTTTACTCATAGGAGTTCCAGGTGTTGTGCTCAT |
| **Hc-aap-gfp-F** | CAGCTCTCCGACCAAATCTGGTACATGAGTAAAGGAGAAGAACTTTTC |
| **Hc-aap-gfp-R** | GAAAAGTTCTTCTCCTTTACTCATGTACCAGATTTGGTCGGAGAGCTG |
| **Ce-age-pstF** | CTGCAGAAGGATCAGAACATCAGAAG |
| **Ce-age-mlbstR** | GTATACACGCGTCTTAAAATCTTAAAATG |
| **Hc-age-mluiF** | ACGCGTATGAGCGGTGCTGCTGTGGCCGCAAGC |
| **Hc-age-bstzR** | GTATACTCAAATGTGCTTGACAGCGTG |
| **Ce-aap-pbamF** | GGA TCC CGA CTT CTC GAG CGA ACA AAG |
| **Ce-aap-pageR** | ACC GGT TCT GAA TCC TTA TAT ATA TAT |
| **Hc-aap-ageF** | ACC GGT ATG GCT GTC GGG CAG CTC TCC |
| **Hc-aap-mluR** | ACG CGT TCA TAA GGC TGG CAT AGT TAG |
| **Hc-age-NdeF** | CATATGATGAGCGGTGCTGCTGTGGCCG |
| **Hc-age-BamR** | GGATCCATCAGTATCCACTTCTTCCGC |
| **Hc-aap-NdeF** | CATATGATGGCTGTCGGGCAGCTCTCC |
| **Hc-aap-BamR** | GGATCCTCATAAGGCTGGCATAGTTAGAG |
