# Supplementary figures and images for: Exploring the role of two interacting phosphoinositide 3-kinases of Haemonchus contortus
Source: Parasit Vectors. 2014 Nov 12;7:498. doi: 10.1186/s13071-014-0498-2 (PMC4233088; doi:10.1186/s13071-014-0498-2)

Additional file 2


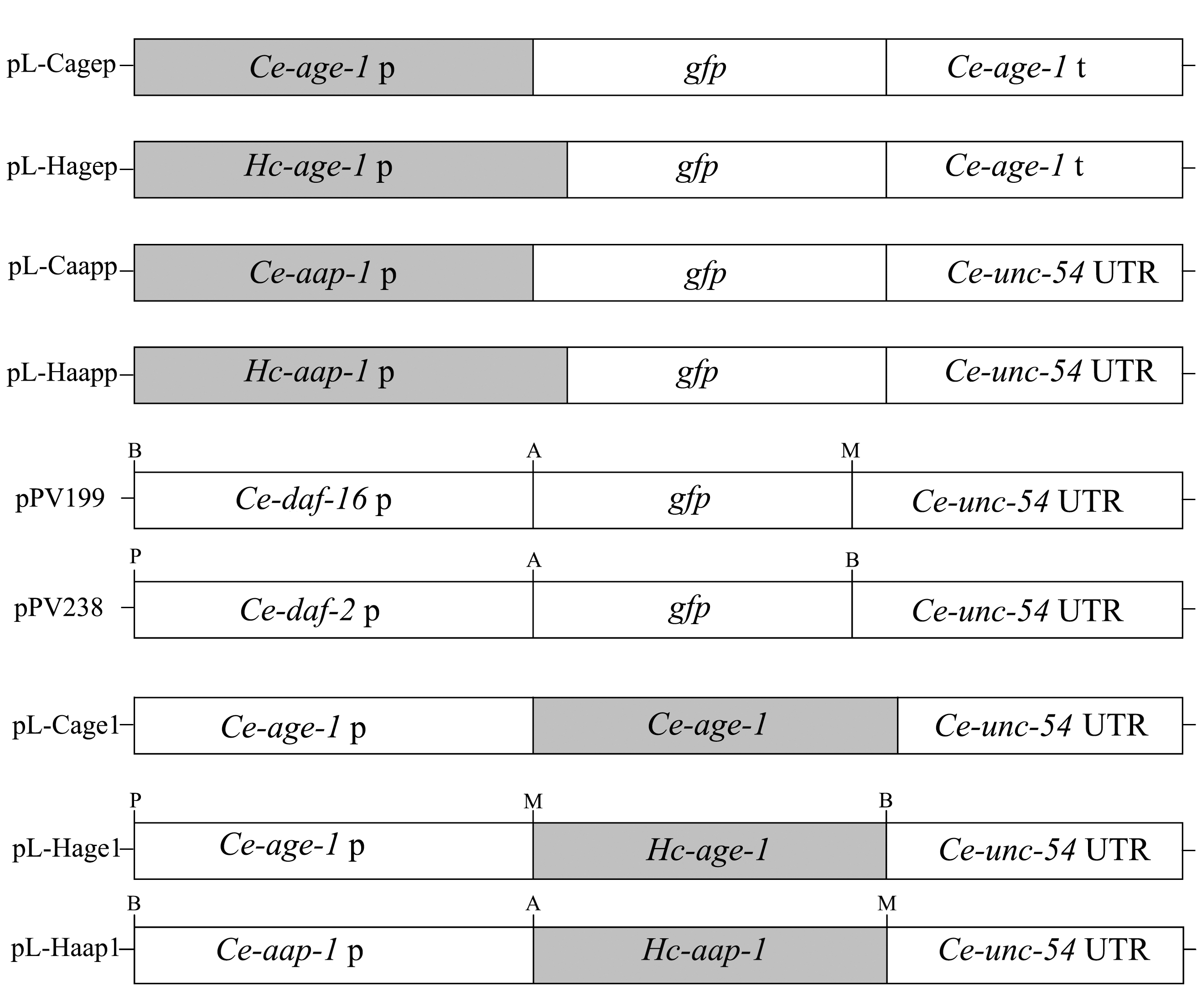

Supplement: Additional file 2: — Cloning strategy for reporter and rescuing constructs. The constructs containing the Caenorhabditis elegans Ce-age-1 and Ce-aap-1 promoters (pL-Cagep and pL-Caapp) and the Haemonchus contortus Hc-age-1 and Hc-aap-1 promoters (pL-Hagep and pL-Haapp) were made based on pPD95.75 by the overlapping extension PCR. The rescuing constructs (pL-Cage1, pL-Hage1 and pL-Haap1) containing the Ce-age-1, Hc-age-1 and Hc-aap-1 coding regions, respectively, were made by removing the gfp coding sequence from pPV238 or pPV199 (cf. 57) and linking the appropriate cDNA. A, B, M and P represent the restriction sites for AgeI, BstZ17I, MluI and PstI, respectively. [file 13071_2014_498_MOESM2_ESM.doc]

Additional file 5


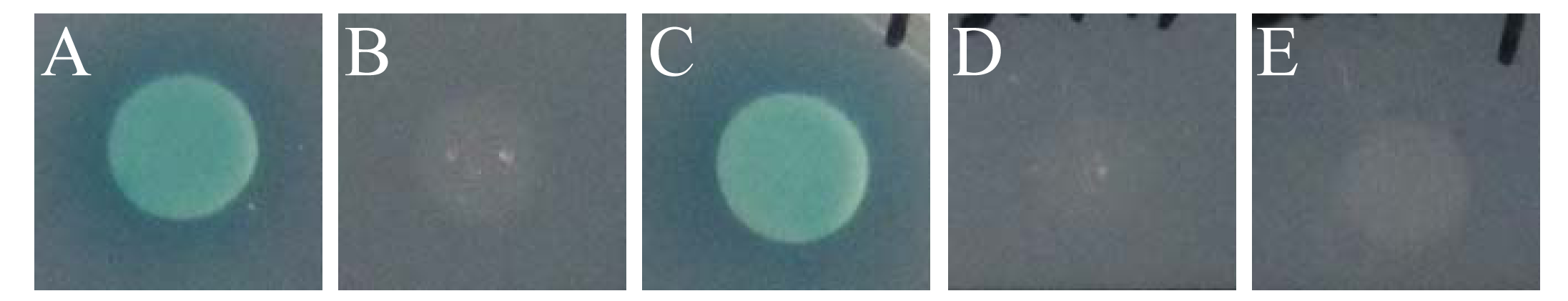

Supplement: Additional file 5: — The adaptor-binding domain of Hc -AGE-1 interacted with Hc -AAP-1 in a yeast two-hybrid assay. (A) The positive control showed that pGBKT7-53 can interact with pGADT7-T; (B) The negative control showed that pGBKT7-Lam and pGADT7-T did not interact; (C) The test group pGBKT7-Hc-age-1 and pGADT7-Hc-aap-1 did interact with QDO/X/A; (D) pGBKT7-Lam and pGADT7-Hc-aap-1 did not interact with QDO/X/A; (E) pGBKT7-Hc-age-1 and pGADT7-T did not interact with QDO/X/A. [file 13071_2014_498_MOESM5_ESM.doc]
