# Supplementary material for: Exploring the role of two interacting phosphoinositide 3-kinases of Haemonchus contortus
Source: Parasit Vectors. 2014 Nov 12;7:498. doi: 10.1186/s13071-014-0498-2 (PMC4233088; doi:10.1186/s13071-014-0498-2)
Supplement: Additional file 3: — The length of exon and intron and splice donor sequences for each exon and intron of Hc-aap-1 gene. The dinucleotide consensus sequence at the splice site is italicized and underlined. All nucleotide sequences are 5’ to 3’. [file 13071_2014_498_MOESM3_ESM.doc]

| Exon no. | Exon size | 5’ splice donor | Intron size | 3’ splice donor |
| --- | --- | --- | --- | --- |
| 1  2  3  4  5  6  7  8  9  10  11 | 69  77  127  127  134  123  114  179  133  72  129 | TTACTGAG*GTGAATAT*  ACCGTCAG*GTTTGTAC*  CACTCAAG*GTCAGTAT*  CAATCCCG*GTGCGTAC*  CTCAAGCT*GTAAGTCA*  ACTCTGAG*GTGAAGCT*  ATGCGAAG*GTTATTCA*  CCGGCAAA*GTAAGTGT*  CTTCACAG*GTAGCCTT*  CCTCACAG*GTGTGTTG*  CCTTATGA*TGTCCCTT* | 77  1890  97  641  1832  74  62  76  57  82 | *TTTCTCAG*ATCATGCA  *CTTTGCAG*ATTTGGTG  *TATTACAG*TGTTACAA  *GTTTTCAG*ATTGGGAA  *ATTTTCAG*ATTGTAGA  *TACTCTAG*CTTGTGGA  *CAATTCAG*GAGCGTCT  *AATTTCAG*CTACCTTG  *GCTTGAAG*GCCAACAA  *TGTTTTAG*GGATGGGG |
