# Supplementary material for: Exploring the role of two interacting phosphoinositide 3-kinases of Haemonchus contortus
Source: Parasit Vectors. 2014 Nov 12;7:498. doi: 10.1186/s13071-014-0498-2 (PMC4233088; doi:10.1186/s13071-014-0498-2)
Supplement: Additional file 4: — The length of exon and intron and splice donor sequences for each exon and intron of Hc-age-1 gene. The dinucleotide consensus sequence at the splice site is italicized and underlined. All nucleotide sequences are 5’ to 3’. [file 13071_2014_498_MOESM4_ESM.doc]

| Exon no. | Exon size | 5’ splice donor | Intron size | 3’ splice donor |
| --- | --- | --- | --- | --- |
| 1  2  3  4  5  6  7  8  9  10  11  12  13  14  15  16  17  18  19  20  21  22  23  24  25  26  27  28  29 | 198  108  92  97  105  106  93  164  90  102  186  93  93  148  125  104  156  97  125  103  75  89  122  116  143  76  194  133  139 | GGAAAAGG*CATGTCGT*  TTCTTACA*GTAAGCTT*  TTGAAAAG*GTATAACT*  ATGAGGAG*GTAAAAAA*  TAGGTCAG*TTACTTCG*  CAGCCGAG*GTTTTTAT*  AATCAAAT*GTTCGTTC*  ACATTAAG*GTGCGATT*  CATATGGG*GTTCGTTG*  TTCACAAG*GTATGTTC*  ATTCACAA*GTTGGTAT*  GGTTGGAA*GTCAGTAC*  AAGGGAAG*GTACGACT*  GAAACAAG*GTTCGTTC*  AGAAGATA*GTGAGTCT*  AACCAAAG*GTAGTTTT*  AGCAAAAA*GTAAGCTA*  TCATTCAA*GTACGTGA*  AATCTCAA*GTGAGTGA*  TGAAACAG*GTCAGCGC*  CTACGGAG*GTGAGTGT*  CACTTGAG*GTTAGTCG*  TGGAGATG*GTAAGTTT*  GCACTAAG*GTATCTCT*  ACGAGAAA*GTGTGTTT*  GAATCATG*GTAGGTTC*  GTTACAAA*GTAAGTAC*  ACCTGAAG*GTTTGCTT*  CATTTGA*ACAGCTTTC* | 293  58  52  330  104  70  187  238  76  1263  88  64  492  581  98  2011  336  676  597  60  392  262  63  451  105  70  682  335 | *CTTTTAGA*GCGACGTT  *GTCTTTAG*GTTTTATT  *TTTTTCAG*AATGTCTG  *GTGTTTAG*ATGCTTTT  *CAGGTCAA*GCGCTTGG  *TCGTTCAG*GTAGCGAA  *CCTTTCAG*CGGCGAAC  *TTGTTTAG*GCGGAAGA  *TCCTTTAG*GAAGTACG  *CCTTACAG*CCCAGTTA  *ACGTTTAG*ATCAGCGT  *TTTTTAAG*GGTATGAT  *TATTTTAG*GCAGAAGA  *AACATCAG*GTTCGAGT  *TTTTTTAG*CAGACCGA  *CGTTATAG*GCATTACG  *TCTTCCAG*ATGCATGG  *CTTTGAAG*GCGCTGAA  *ATGTTTAG*AGATGCGG  *TATTGTAG*GTGGAAAT  *AATTACAG*CGCCTTCG  *TTTTATAG*GATCGACA  *GCTTTCAG*ATCTGCGC  *CATTTCAG*TATGGCAT  *CCTTGTAG*GTATATGG  *ATTCGCAG*GATCGGCA  *TACTTCAG*ATTCAAAA  *TTTTTTAG*AGCACCCT |
